# Decoupled Bidirectional Spatio-Temporal Fusion Network for Hybrid EEG-fNIRS Cognitive Task Classification

**DOI:** 10.3390/brainsci16020241

**Published:** 2026-02-21

**Authors:** Zirui Wang, Guanghao Huang, Zhuochao Chen, Xiaorui Liu, Yinhua Liu, Keum-Shik Hong

**Affiliations:** 1 Institute for Future, School of Automation, Qingdao University, Qingdao 266071, China; wangzirui34@qdu.edu.cn (Z.W.); chenzhuochao@qdu.edu.cn (Z.C.); liuxiaorui@qdu.edu.cn (X.L.); liuyinhua@qdu.edu.cn (Y.L.); ksh@qdu.edu.cn (K.-S.H.); 2 School of Mechanical Engineering, Pusan National University, Busan 46241, Republic of Korea

**Keywords:** multimodal neuroimaging, EEG, fNIRS, spatio-temporal alignment, decoupled fusion, cognitive task classification

## Abstract

**Highlights:**

**What are the main findings?**

- A novel decoupled deep learning framework, BiSTF-Net, is proposed, which systematically addresses the spatio-temporal heterogeneity between EEG and fNIRS signals through bidirectional spatial guidance and adaptive temporal alignment.
- The framework introduces a decoupled, multi-stage fusion pipeline, featuring a bi-directional cross-modal guidance (Bi-CMG) module for early spatial feature enhancement and a symmetric cross-attention fusion (SCAF) module for late-stage deep fusion.

**What are the implications of the main findings?**

- The proposed adaptive temporal alignment (ATA) module provides a data-driven solution to overcome the critical bottleneck of inherent, subject-specific delays in fNIRS signals, enhancing the reliability and accuracy of multimodal data.
- This work offers a powerful and interpretable paradigm for multimodal neural classification, whose decoupled design (Bi-CMG, ATA, SCAF) provides an effective and generalizable solution for tackling heterogeneous signal fusion challenges in neuroengineering.

**Abstract:**

**Background/Objectives:** Multimodal neuroimaging, particularly the integration of electroencephalography (EEG) and functional near-infrared spectroscopy (fNIRS), has emerged as a key methodology for investigating brain function and classifying neural activity. However, the efficient fusion of these two signals remains a formidable challenge due to their significant spatio-temporal heterogeneity. This paper presents the BiSTF-Net, which integrates decoupled and bi-directional spatio-temporal fusion mechanisms to enhance the performance of cognitive task recognition. **Methods**: In BiSTF-Net, the spatial features of EEG and fNIRS are mutually guided and enhanced through an efficient bi-directional cross modal guidance (Bi-CMG). Then, the temporal latencies of fNIRS signals are aligned in a data-driven manner using adaptive temporal alignment (ATA). Subsequently, the aligned features are deeply fused into a modality-invariant, discriminative representation via a symmetric cross-attention fusion (SCAF) module. **Results**: Evaluated on the mental arithmetic (MA), motor imagery (MI), and word generation (WG) tasks, the BiSTF-Net achieves average accuracies of 83.33%, 82.09%, and 84.99% respectively. **Conclusions**: The BiSTF-Net exhibits superior performance compared to the existing methods, offers a robust and interpretable solution for multimodal EEG-fNIRS cognitive task classification, and provides a methodological foundation for future extensions to other multimodal data and broader real-world clinical applications.

## 1. Introduction

Neuroimaging techniques provide a crucial window into understanding brain activity. By classifying neural signals, these technologies have demonstrated significant potential in areas such as medical rehabilitation [1], neurodiagnostics [2,3,4,5], and human–computer interaction [6]. Electroencephalography (EEG) [7,8,9,10] and functional near-infrared spectroscopy (fNIRS) [11,12,13,14,15] have garnered more extensive research and attention due to their cost-effective, portability, and ease of use, especially when compared to magnetoencephalography (MEG) [16,17] and functional magnetic resonance imaging (fMRI) [18,19]. EEG offers high temporal resolution in the millisecond range, enabling precise capture of fast neural electrical activity. However, its spatial resolution is relatively low due to signal diffusion through the skull and tissues, and it is susceptible to motion artifacts and electrical noise interference. In contrast, fNIRS indirectly reflects neural activity through changes in oxygenated hemoglobin (HbO) and deoxygenated hemoglobin (HbR) concentration as a hemodynamic response. fNIRS offers higher spatial resolution than EEG and exhibits strong resistance to interference. However, its temporal resolution is comparatively lower, and it suffers from signal delay issues. Therefore, relying solely on EEG or fNIRS for neural signal acquisition has inherent limitations in information dimensionality, making it difficult to fully and accurately capture the dynamic changes in brain activity.

To overcome the inherent limitations of single modalities and fully exploit their complementary advantages in spatial and temporal characteristics, EEG-fNIRS multimodal fusion approaches are widely explored [20,21,22,23]. Numerous studies have confirmed that classifying schemes based on EEG-fNIRS fusion significantly outperform single-modality methods using only EEG or fNIRS in terms of both accuracy and stability [24,25]. Since EEG and fNIRS do not interfere with each other in terms of technical principles and are highly compatible in hardware [26,27], combining these two modalities into a multimodal neuroimaging system has become an attractive research direction. This fusion strategy allows for the simultaneous capture of rapid dynamic neural changes (from EEG) and enhanced spatial sensitivity (from fNIRS). The complementary and corroborative nature of the two signals not only reduces the potential for misinterpretation that may arise from single-modality analysis but also makes it particularly suitable for classifying complex cognitive tasks that require high spatio-temporal precision, such as motor imagery [22,28], mental arithmetic [29] and word generation [30].

Most traditional EEG-fNIRS fusion research focuses on feature- or decision-level fusion techniques, which aim to improve performance by simply combining manually extracted features or decision scores. Feature-level fusion involves manually extracting time–frequency features from EEG and changes in HbO concentration from fNIRS, followed by inputting these features into a classifier for joint training. For instance, one common strategy is to use EEG data to guide the extraction of fNIRS features, which are then combined with spatial features from the EEG signal itself for classification [25,31]. Further advancing feature fusion, some methods create a unified spatio-temporal feature representation, such as using a (2+1) D architecture to organize EEG and fNIRS signals into a single 4D tensor for joint processing [32]. Decision-level fusion involves separately training classification models for EEG and fNIRS, and then integrating their predictions through weighted voting or a meta-classifier. For instance, a maximum likelihood estimation is employed to integrate the decisions from various classification network combinations, evaluating them across multiple tasks [33]. Another approach extracts prediction scores from EEG and fNIRS signals and uses a linear discriminant analysis (LDA)-based meta-classifier to obtain the final prediction score [34].

In recent years, deep learning has provided new avenues for addressing the above challenges [35]. Data-driven methods, such as cross-attention [35,36], have demonstrated the feasibility of adaptive temporal alignment. However, existing fusion strategies still generally suffer from insufficient interactivity and a lack of guidance in the fusion process [37,38]. To address these issues, sophisticated architectures that perform successive fusion at both feature and decision levels [39], attention mechanisms that dynamically guide the fusion process [40,41,42], multitask learning (MTL) to learn more discriminative representations [43], and novel end-to-end deep learning models (EF-Net) to improve generalization to unseen subjects [44] have been developed. While these results have provided strong impetus for the evolution of multimodal fusion methods, practical challenges remain, including insufficient temporal synchronization between modalities and inadequate interactivity across modalities.

To benchmark our proposed framework, we selected several state-of-the-art methods for comparison. These include advanced deep learning models such as STA-Net and E-FNet, which also utilize attention mechanisms for multimodal fusion. STA-Net primarily employs spatial-temporal alignment network for feature extraction, while E-FNet focuses on a dual-stream architecture. We also compare our method with simpler approaches such as ST2A and M2NN, which represent feature and decision-level fusion strategies respectively. While these methods have demonstrated contributions in specific areas, they often rely on simplified fusion mechanisms or lack comprehensive spatio-temporal alignment strategies, which limit their performance in handling the complex heterogeneity of EEG-fNIRS signals.

In this paper, a novel decoupled bidirectional spatio-temporal fusion network (BiSTF-Net) is proposed for classification the hybrid EEG-fNIRS signals. The BiSTF-Net systematically addresses the heterogeneity between EEG and fNIRS signals through three core components: hierarchical feature extraction, decoupled spatio-temporal fusion, and multi-task classifiers. It achieves more precise time synchronization and deeper Bidirectional feature fusion to enhance classification performance. The main contributions of this study are summarized as follows:

(1) A bi-directional cross-modal guidance module (Bi-CMG) employs the bidirectional cross-attention mechanism to facilitate mutual guidance and enhancement of the spatio-temporal features between EEG and fNIRS.

(2) An adaptive temporal alignment (ATA) module employs a bidirectional gated recurrent unit (Bi-GRU) enhanced attention mechanism to achieve dynamic synchronization by aligning fNIRS’s hemodynamic patterns with corresponding EEG features.

(3) A symmetric cross-attention fusion (SCAF) module is employed for deep multimodal fusion, which learns a modality-invariant representation through a symmetric interaction mechanism and a consistency loss constraint.

## 2. Methods

**Figure 1:**
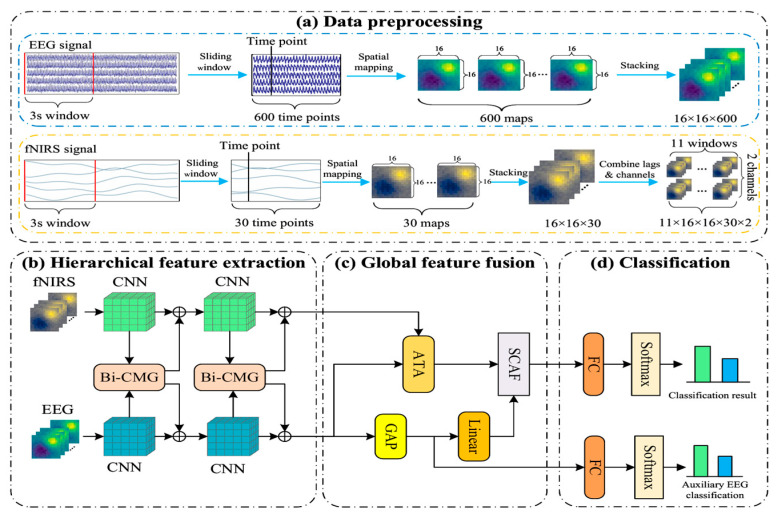
The overall framework of the proposed BiSTF-Net. The framework comprises four stages: (**a**) Data preprocessing. (**b**) Hierarchical feature extraction, where parallel CNNs extract features enhanced by Bi-CMG (bi-directional cross-modal guidance) modules. (**c**) Global feature fusion using ATA (adaptive temporal alignment) module for temporal alignment and SCAF (symmetric cross-attention fusion) module for deep fusion, where GAP refers to global average pooling. (**d**) Classification.

To address the spatio-temporal heterogeneity challenge in EEG and fNIRS signals, we propose a novel decoupled BiSTF-Net, as illustrated in Figure 1. In initial stage of the BiSTF-Net, continuous multi-channel signals are first segmented using a 3-s sliding window. Each resulting sample is then transformed into a high-dimensional spatio-temporal tensor by converting the multi-channel data at each time point into a 16 × 16 spatial map via interpolation, and subsequently stacking these maps along the time axis.

Following the preprocessing stage, the model proceeds through three core components for deep fusion. First, in the hierarchical feature extraction stage, modality-specific 3D CNNs extract features that are mutually enhanced by Bi-CMG modules. Second, during global feature fusion, the ATA module performs adaptive temporal alignment while the SCAF module conducts deep fusion. Finally, the classification stage utilizes a multi-task head to generate the final prediction.

### 2.1. The Bi-CMG Module

**Figure 2:**
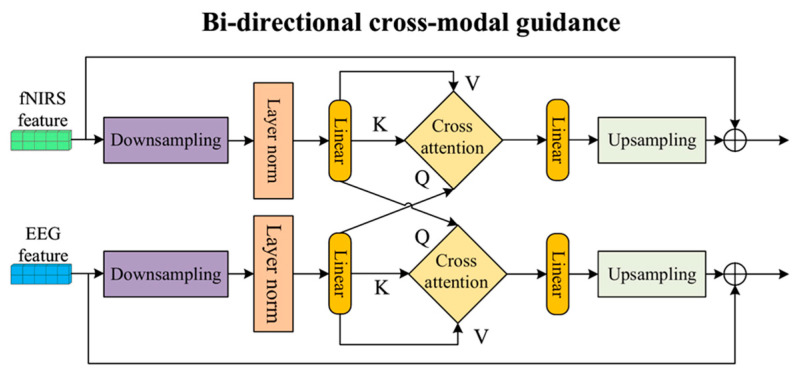
The detailed architecture of the Bi-CMG (bi-directional cross-modal guidance) module.

To facilitate early cross-modal spatial interaction, a Bi-CMG module is embedded in each feature extraction unit, as shown in Figure 2. The Bi-CMG is designed to achieve mutual guidance and enhancement of the spatial feature maps from EEG and fNIRS at each hierarchical level through an efficient cross-attention mechanism. High-resolution feature maps are first spatially downsampled to generate computationally lighter surrogate features. The guidance information obtained from the surrogate features is then upsampled and applied back to the original feature maps. In this way, the Bi-CMG module ensures computational efficiency while effectively enabling cross-modal spatial guidance.

Specifically, for the input EEG feature map $F_{EEG}\in R^{B\times C_{e}\times D_{e}\times H_{e}\times W_{e}}$ and fNIRS feature map $F_{fNIRS}\in R^{B\times C_{f}\times D_{f}\times H_{f}\times W_{f}}$, where B is the batch size, C denotes the number of channels, D represents the temporal depth, and H and W are the spatial height and width, respectively. The module generates low-resolution surrogate features PEEG and PfNIRS through adaptive average pooling to significantly reduce the complexity of subsequent attention computations. In the path where fNIRS guides EEG, the EEG surrogate features, after layer normalization, are linearly mapped into a query matrix $Q_{EEG}\in R^{B\times N\times 64}$, while the fNIRS surrogate features are linearly mapped into key matrix $K_{fNIRS}\in R^{B\times N\times 64}$ and value matrix $V_{fNIRS}\in R^{B\times N\times 64}$, and vice versa, where N represents the sequence length of the surrogate feature tokens after pooling, and 64 is the feature dimension of the attention mechanism. These matrices are then input into a multi-head cross-attention unit, and the computation process can be expressed as:

(1) $$\mathrm{Attention}(Q_{\mathrm{EEG}},K_{\mathrm{fNIRS}},V_{\mathrm{fNIRS}})=\mathrm{softmax}\left(\frac{Q_{\mathrm{EEG}}K_{\mathrm{fNIRS}}^{T}}{\sqrt{d_{k}}}\right)V_{\mathrm{fNIRS}},$$

where d_k_ = 16 is the dimension of each attention head. This mechanism dynamically retrieves fNIRS features by leveraging EEG features, generating contextual guidance information for the current EEG features through a weighted aggregation of fNIRS data based on relevance scores. The guidance information is then transformed linearly via a linear layer and upsampled to the resolution of the original feature map using trilinear interpolation. Finally, the upsampled guidance information is added to the original EEG feature map through a residual connection, resulting in the enhanced features. This residual structure helps mitigate the vanishing gradient problem and ensures the stability of information flow.

### 2.2. The ATA Module

**Figure 3:**
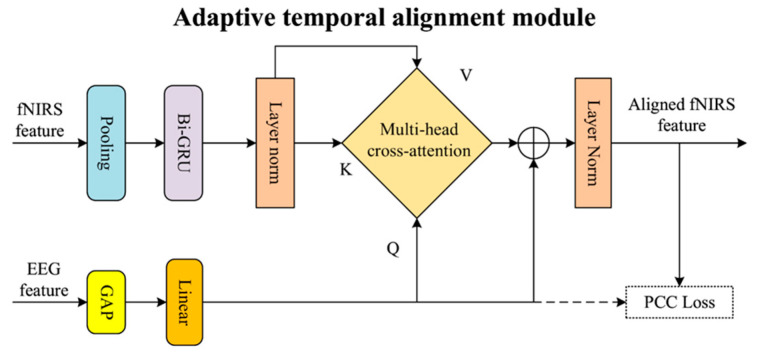
The detailed architecture of the ATA (adaptive temporal alignment) module.

After the initial feature interaction, the primary challenge for the model is to account for the inherent and subject-specific hemodynamic delay in fNIRS signals. To address this, an ATA module is designed, with its detailed architecture shown in Figure 3. The key idea of this module is to use high-temporal-resolution EEG features as a temporal anchor, dynamically localizing and aggregating the most relevant portions of the fNIRS feature sequence, thereby achieving adaptive temporal alignment.

The ATA module operates in a data-driven fashion, leveraging an attention mechanism combined with a Pearson correlation coefficient (PCC) loss to dynamically discover temporal alignments between EEG and fNIRS features. While our approach does not explicitly model the canonical HRF convolution, which is a common strategy to predict fNIRS latencies from EEG events, its data-driven nature allows for more flexible and adaptive discovery of latent temporal relationships that might be more suitable for complex cognitive tasks.

To facilitate this data-driven alignment, for each 3-s EEG feature block (serving as a temporal anchor), the ATA module considers a search space of 11 overlapping 3-s fNIRS feature blocks, forming a 10-s statistical alignment window. This design ensures that the module can effectively capture and align fNIRS hemodynamic responses that typically occur within the expected neurophysiological latency range of approximately 4–7 s after an EEG event.

To clarify the role of global average pooling (GAP) and linear projection within the ATA module, especially regarding the ‘high-temporal-resolution EEG features as a temporal anchor,’ we emphasize the following. The GAP operation is applied across the spatial dimensions (Height and Width) of the advanced EEG features at each individual time point, creating a compact spatial summary for that moment. Critically, GAP does NOT pool along the temporal dimension. The subsequent linear projection then transforms this spatial summary into a query vector, QEEG. The term ‘high-temporal-resolution EEG features as a temporal anchor’ refers to the fact that each QEEG vector corresponds to a specific, high-resolution time point of the original EEG signal. Therefore, the sequence of these QEEG vectors collectively maintains the high temporal fidelity of the EEG features, acting as a series of distinct semantic-level temporal anchors. This sequence guides the adaptive search for corresponding fNIRS patterns across its 10-s search space. The primary objective is to extract high-level semantic guidance for temporal synchronization, rather than preserving the raw, low-level time–frequency details of the original EEG signal in the query itself.

The module receives an EEG feature block $F_{EEG}\in R^{B\times 16\times 4\times 4\times 50}$ and an fNIRS feature block $F_{fNIRS}\in R^{B\times 16\times 4\times 4\times 50}$ as input from the second feature extraction layer. Internally, the depth dimension of the fNIRS feature block (size 4) is treated as a temporal sequence and is innovatively encoded using a Bi-GRU to capture its intrinsic dynamic evolution patterns. Meanwhile, the instantaneous EEG feature block is processed through global average pooling and linear projection to form a query vector QEEG. Subsequently, the model performs multi-head cross-attention computation, where the EEG query vector guides weighted information extraction from the Bi-GRU-encoded fNIRS feature sequence. In this process, the fNIRS feature sequence serves as both the key and the value. This computation can be expressed as:

(2) $$F_{\mathrm{fNIRS}}^{\prime }=\mathrm{softmax}\left(\frac{Q_{\mathrm{EEG}}\cdot H_{\mathrm{fNIRS}}^{T}}{\sqrt{d_{k}}}\right)H_{\mathrm{fNIRS}},$$

where $F_{fNIRS}^{\prime }$ denotes the temporally aligned fNIRS feature, $Q_{EEG}$ represents the query vector derived from EEG features acting as a temporal anchor, $H_{fNIRS}$ is the fNIRS feature sequence encoded by the Bi-GRU (serving as both the key and value).

The output of the attention computation is passed through a residual connection and layer normalization, resulting in an fNIRS feature vector $F_{fNIRS}^{\prime }\in R^{B\times 256}$, which is precisely temporally aligned with the EEG signal. To ensure the effectiveness of this alignment, the entire module is trained under the supervision of a Pearson correlation coefficient loss (PCC Loss), which aims to maximize the linear correlation between the EEG query vector and the final output fNIRS features. The loss is defined as:

(3) $$L_{\mathrm{PCC}}=-E\left[\frac{\left(Q_{\mathrm{EEG}}-\mu_{Q})(F_{\mathrm{fNIRS}}^{\prime }-\mu_{F^{\prime }}\right)}{\sigma_{Q}\sigma_{F^{\prime }}}\right],$$

where µ and σ represent the mean and standard deviation, respectively. The loss guides the model to learn synchronization based on semantics rather than pure numerical alignment.

### 2.3. The SCAF Module

**Figure 4:**
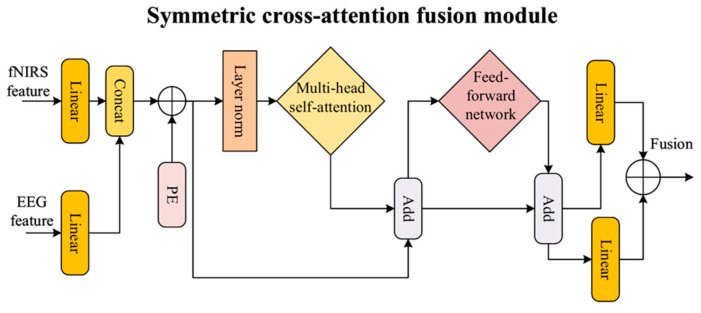
The detailed architecture of the SCAF (symmetric cross-attention fusion) module.

After completing the temporal alignment, a SCAF module is designed for the final deep fusion, as shown in Figure 4. The core of this module is to perform deep interaction and fusion of the high-level features from both modalities within an entirely equal framework, aiming to learn a modality-invariant and more robust fused feature.

This deep fusion is predicated on the understanding that while EEG and fNIRS capture distinct neurophysiological processes (electrical activity vs. hemodynamic response), they provide complementary information about the same underlying cognitive events. Therefore, the SCAF module’s design does not aim to force one modality’s representation to numerically resemble the other at a raw signal or low-level feature stage. Instead, its core philosophy is to distill a unified, richer representation where the unique strengths of each modality are integrated in a semantically consistent and modality-invariant manner within a higher-level abstract feature space. This approach leverages the synergistic interaction to yield a more robust and discriminative fused feature.

The module receives two feature vectors with dimensions of R^B×256^, one is the aligned fNIRS feature output from the ATA, and the other is the EEG feature after linear projection. Inside the module, both input vectors are first mapped to a 64-dimensional feature space via independent linear layers. Then, they are concatenated along the sequence dimension to form a tensor of sequence length 2, with shape R^B×2×64^, where the two elements of the sequence represent the EEG and fNIRS modalities, respectively. To incorporate positional information into this sequence, a learnable positional encoding matrix P∈R^1×2×64^ is added to the tensor.

After the positional encoding is added, the sequence tensor X is fed into a transformer-based BiFormer unit. This unit employs a multi-head self-attention mechanism, where the input is processed by multiple parallel attention heads. The computation for a single head is defined as:

(4) $$\mathrm{Attention}(Q,K,V)=\mathrm{softmax}\left(\frac{QK^{T}}{\sqrt{d_{k}}}\right)V.$$

The outputs of all heads are then concatenated and linearly projected to produce the final output of the multi-head attention layer:

(5) $$\mathrm{MultiHeadAttention}(X)=\mathrm{Concat}({\mathrm{head}}_{1},\ldots ,{\mathrm{head}}_{h})W^{O},$$

where ${head}_{i}=Atention({XW}_{i}^{Q},{XW}_{i}^{K},{XW}_{i}^{V})$,and $W_{i}^{Q}$, $W_{i}^{K}$, $W_{i}^{V}$, and W^O^ are learnable parameter matrices. A standard transformer encoder block consists of this multi-head self-attention sub-layer followed by a position-wise feed-forward network (FFN). Layer normalization and residual connections are applied after each sub-layer. The output of the block is computed in two sequential steps:

(6) $$X^{\prime }=\mathrm{LN}(X+\mathrm{MultiHeadAttention}(X)),$$

(7) $$F_{\mathrm{SCAF}}=\mathrm{LN}(X^{\prime }+\mathrm{FFN}(X^{\prime })),$$

where X is the input tensor to the block, X’ is the output of the first sub-layer, and F_SCAF_ is the final output. Through this mechanism, the feature representations of EEG and fNIRS undergo deep information interaction, enabling each modality to effectively integrate complementary information from the other. The resulting sequence is then separated back into independent EEG representation $F_{EEG}\in R^{B\times 64}\;$ and fNIRS representation $F_{fNIRS}\in R^{B\times 64}$. These two features are subsequently refined through separate linear projection layers, producing the fused features $F_{EEG}^{\prime }$ and $F_{fNIRS}^{\prime }$. To encourage greater consistency between their representations, a mean squared error (MSE) loss is introduced as a supervisory signal:

(8) $$L_{\mathrm{MSE}}=\frac{1}{BD}\sum_{i=1}^{B}\sum_{j=1}^{D}{\left(F_{\mathrm{EEG},ij}^{\prime }-F_{\mathrm{fNIRS},ij}^{\prime }\right)}^{2},$$

This loss function forces the refined features from both modalities to converge numerically. Finally, these two feature vectors are fused through element-wise addition, generating the final fused feature *F*_fused_∈R^B×64^, which serves as the sole input for the main classification path.

### 2.4. Loss Function

To ensure the synergy of all modules, a comprehensive joint loss function L_total_ is designed. This function consists of the main classification loss term L_main_, the auxiliary classification loss term L_aux_, and two regularization loss terms, L_PCC_ and L_MSE_, which supervise the ATA and SCAF modules, respectively, with appropriate weighting.

(9) $$L_{\mathrm{total}}=w_{1}L_{\mathrm{main}}+w_{2}L_{\mathrm{aux}}+w_{3}L_{\mathrm{PCC}}+w_{4}L_{\mathrm{MSE}},$$

where w_1_, w_2_, w_3_, w_4_ are hyper-parameters used to balance the importance of each term. The main classification loss L_main_ is the core driving force of the model’s training, and it uses the standard cross-entropy loss to measure the accuracy of the model’s classification on the final fused features, defined as:

(10) $$L_{\mathrm{main}}=-\frac{1}{B}\sum_{i=1}^{B}\sum_{c=1}^{N_{\mathrm{class}}}y_{ic}\log({\hat{y}}_{ic}),$$

where N_class_ is the number of classes, $y_{\mathrm{ic}}$ is the true label, and ${\hat{y}}_{\mathrm{ic}}$ is the predicted probability from the main classifier. The auxiliary classification loss L_aux_ also uses cross-entropy loss, but it only applies to the independent EEG branch and serves as a regularization method to ensure that the branch independently learns effective discriminative information, defined as:

(11) $$L_{\mathrm{aux}}=-\frac{1}{B}\sum_{i=1}^{B}\sum_{c=1}^{N_{\mathrm{class}}}y_{ic}\log({\hat{y}}_{ic}^{\mathrm{EEG}}),$$

where ${ŷ}_{ic}^{EEG}$ denotes the predicted probability generated by the auxiliary classifier. The PCC loss, defined in Equation (3), supervises the temporal alignment process of the ATA module by maximizing the Pearson correlation between the EEG query vector and the aligned fNIRS feature vector. This approach guides the ATA to learn a meaningful, semantically based synchronization rather than a purely numerical one. This loss guides the ATA to learn a meaningful, semantically based synchronization rather than a purely numerical one

Finally, the MSE loss, as defined in Equation (8), supervises the feature consistency learning process. Addressing the reviewer’s concern regarding enforcing numerical similarity between distinct neurophysiological processes, it is crucial to clarify that the LMSE operates on the refined, high-level features (FEEG and FfNIRS) after they have undergone symmetric cross-attention fusion within the SCAF module. The objective is not to force EEG’s raw temporal and spatial characteristics to resemble fNIRS’s, nor vice versa, as these signals indeed represent distinct physiological phenomena. Instead, the LMSE aims to encourage semantic consistency and modality-invariance in the abstract feature space derived from the symmetric interaction. By minimizing the discrepancy between the refined EEG and fNIRS feature representations, the loss guides the model to learn a unified, modality-agnostic representation where complementary information from both sources is integrated robustly. This ensures that the fused features reliably reflect the underlying cognitive state, regardless of modality-specific variations, thereby enhancing the stability and discriminative power of the final representation without conflating the distinct biophysical origins of the signals. Leveraging a joint loss function enables the collaborative optimization of all components within a unified framework. This approach not only ensures strong final classification performance but also effectively safeguards the validity and robustness of intermediate processes, such as temporal alignment and feature fusion.

## 3. Experiments and Results

### 3.1. Dataset Description

For the experiments, two EEG-fNIRS datasets released by Shin et al. from the Berlin Institute of Technology were used. The first dataset [34], used for the MI and MA tasks, contains recordings from 29 healthy participants (mean age 28.5 ± 3.7 years). It provides EEG signals from 30 channels and fNIRS signals from 36 channels (14 sources, 16 detectors). The original sampling rates were 1000 Hz (EEG) and 12.5 Hz (fNIRS).

The second dataset [45], used for the WG task, contains synchronized data from 26 right-handed healthy subjects (mean age 26.1 ± 3.5 years). This dataset provides 28 channels of EEG data (from a 30-electrode setup where 2 were used for reference/ground) and 36 channels of fNIRS data. The original sampling rates were 1000 Hz (EEG) and 10.4 Hz (fNIRS). For our model validation, only the WG task was selected.

The MI and MA tasks from the first dataset [34] followed a trial structure, consisting of a 2-s cue, a 10-s task execution phase, and a 15–17 s rest period. The WG task from the second dataset [45] followed similar trial structure, consisting of a 2 s of instructions, a 10 s of task execution, and a 13–15 s of rest.

### 3.2. Data Preprocessing

The multi-channel EEG and fNIRS signals from the two datasets were first downsampled to a sampling rate of 200 Hz and 10 Hz, respectively, and processed via a unified pipeline using the MNE-Python software package (version 1.8.0) and Python (version 3.9.21). For EEG signals, after downsampling, a specific 50 Hz notch filter was first applied to precisely remove the prominent power-line interference. Subsequently, an IIR (Infinite Impulse Response) Butterworth bandpass filter of order 6 was applied with cut-off frequencies between 0.5 Hz and 50 Hz, noting that the 50 Hz component had already been effectively attenuated by the preceding notch filter. Following filtering, common average re-referencing was performed. To further mitigate artifacts, Independent Component Analysis (ICA) was utilized. ICA components highly correlated with known artifactual sources (e.g., eye blinks, muscle activity) were identified through visual inspection of their time courses and topographies and subsequently removed. For fNIRS signals, after downsampling, HbO/HbR concentration changes were computed using the modified Beer–Lambert law. These signals were then bandpass filtered using an IIR Butterworth filter of order 6, with cut-off frequencies between 0.01 Hz and 0.1 Hz. Baseline correction was applied to each trial using the pre-task average (from trial onset to 3 s post-onset) as a reference.

**Figure 5:**
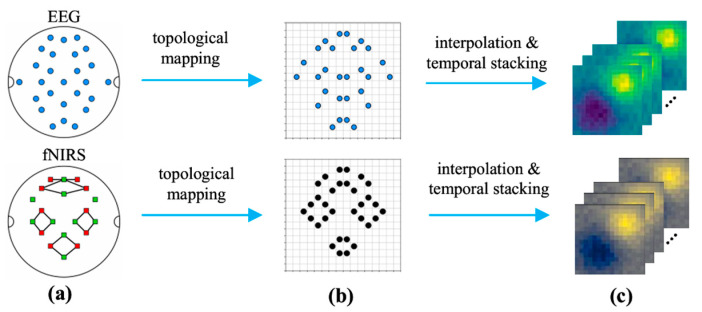
Conceptual illustration of the spatio-temporal tensor construction. (**a**) Layouts of EEG (**top**) and fNIRS (**bottom**) sensors on the scalp. (**b**) Topological mapping of sensor positions to a 16 × 16 grid. (**c**) Final spatio-temporal tensors formed after interpolation and temporal stacking.

Subsequently, model input samples were constructed through four key steps. First, EEG trials were segmented into 15-s windows (5 s pre-task + 10 s post-task) to capture neural responses, and fNIRS trials into 27-s windows (5 s pre-task + 22 s post-task) to account for delayed hemodynamic responses. Second, a 3-s sliding window with a 1-s step was applied to each trial for data augmentation, increasing the number of training instances. Third, to address fNIRS hemodynamic delay, a data-driven cross-modal temporal alignment strategy was adopted. For each 3-s EEG sample serving as a temporal anchor, 11 overlapping 3-s fNIRS windows were considered (forming a 10-s search space) to cover the typical response latency (4–6 s), enabling dynamic correlation with EEG neural activity [37]. Finally, channel signals were mapped to 16 × 16 grid-based high-dimensional tensors via sensor layout schematic, topological mapping, and interpolation stacking, as shown in Figure 5.

For EEG, the channel-based data within each 3-s sliding window sample was projected onto a 16 × 16 grid at each time point using bi-dimensional cubic spline interpolation. Any remaining NaN values after cubic interpolation were filled using nearest neighbor interpolation. These 600 spatial maps (one for each time point in a 3-s EEG window sampled at 200 Hz) were then stacked, generating a 3D spatio-temporal tensor $X_{EEG}\in R^{16\times 16\times 600}$. To prepare this for the network, its dimensions were permuted to [1 × 600 × 16 × 16] (Channel × Depth × Height × Width), serving as a direct input to the 3D CNN which expects a single channel.

For fNIRS, a similar spatial mapping was performed for each of the 30 time points within each of the 11 windows, considering both HbO and HbR. This process formed a five-dimensional tensor $X_{fNIRS}\in R^{11\times 16\times 16\times 30\times 2}$. To adapt this 5D tensor for the 3D CNN architecture, a critical reshaping step is performed within the model’s forward pass: the window dimension (11) and the chromophore dimension (2) are collapsed into a single channel dimension. This results in a final input tensor of shape [22 × 30 × 16 × 16] (Channels × Depth × Height × Width), which is then fed into the fNIRS CNN pathway.

### 3.3. Experimental Setup and Evaluation Metrics

#### 3.3.1. Experimental Setup

All experiments were implemented using the PyTorch (version 2.6.0) framework on a computing platform equipped with NVIDIA GPUs. To ensure result reproducibility, a global random seed was set to 42, and cuDNN’s benchmark mode was enabled to optimize computational efficiency. The AdamW optimizer [46] was used for model training, with an initial learning rate of 5 × 10^−4^ and weight decay of 10^−2^. The learning rate was smoothly decayed to 10^−6^ over 200 epochs using a cosine annealing strategy. The batch size was set to 64, with early stopping implemented, terminating training if the validation accuracy did not improve for 30 consecutive epochs. The weights for the individual loss components of the joint loss function were set as follows: The weight for the main classification loss w_1_ = 2.0, the weight for the auxiliary EEG classification loss w_2_ = 1.0, the weight for the PCC loss of the ATA module w_3_ = 0.2, The weight for the MSE loss of the SCAF module w_4_, adopted a dynamic warm-up strategy, linearly increasing from 0 to 1.0 over the first 50 epochs. This strategy aimed to allow the model to focus initially on the classification task and gradually introduce stronger constraints for modality consistency once the model stabilized.

#### 3.3.2. Evaluation Scheme and Metrics

To obtain reliable and unbiased performance evaluations, a 10-fold cross-validation scheme was employed for each subject’s data. This scheme randomly divides all trials of each subject into 10 mutually exclusive subsets, ensuring class distribution proportionality. Nine of these subsets are used for training, and one is used for testing in each fold. The final performance for each subject is reported as the average of the 10-fold test results. The model’s classification performance was evaluated using four widely used classification metrics: accuracy, precision, recall, and F1-score.

(12) $$\mathrm{Accuracy}=\frac{TP+TN}{TP+TN+FP+FN},$$

(13) $$\mathrm{Precision}=\frac{TP}{TP+FP},$$

(14) $$\mathrm{Recall}=\frac{TP}{TP+FN},$$

(15) $$F1-\mathrm{Score}=2\frac{\left(\mathrm{Precision}\cdot \mathrm{Recall}\right)}{\left(\mathrm{Precision}+\mathrm{Recall}\right)}.$$

Accuracy represents the proportion of correctly classified samples out of the total samples, where TP, TN, FP, and FN denote the numbers of true positives, true negatives, false positives, and false negatives, respectively. Precision measures the proportion of actual positive samples among all samples predicted as positive. Recall evaluates the proportion of successfully predicted positive samples out of all actual positive samples. The F1 score represents the harmonic mean of precision and recall, providing a balanced assessment of the model’s performance, especially in the case of class imbalance. In this study, precision, recall, and F1 score were calculated using macro averaging. The final performance for all subjects is presented as the average of these metrics in the Section 3.

### 3.4. Overall Classification Performance

The overall classification performance of the proposed BiSTF-Net was evaluated. The results demonstrate robust performance across all three cognitive tasks, with average accuracies reaching 83.33%, 82.09%, and 84.99% for MA, MI, and WG tasks, respectively.

**Figure 6:**
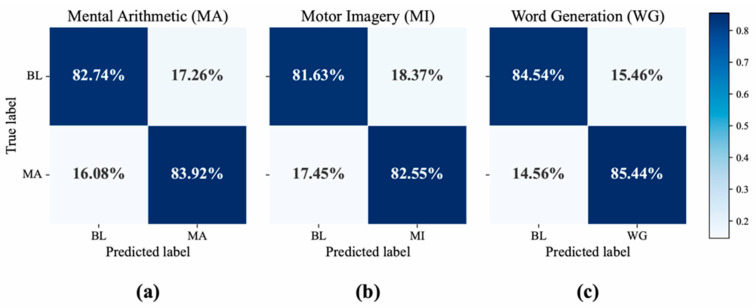
Confusion matrices obtained using the BiSTF-Net (BL: baseline). (**a**) Mental arithmetic (MA); (**b**) Motor imagery (MI); (**c**) Word generation (WG) tasks.

To illustrate the model’s specific classification behavior, confusion matrices for the three tasks are presented in Figure 6. The results demonstrate consistently high performance across all three tasks. The strong diagonal values indicate high recall for both the baseline and the task-specific classes. For instance, in the MI task, the model correctly identified the baseline and MI states with 83% and 81% recall, respectively, while maintaining low misclassification rates. This robust performance across different cognitive tasks highlights the model’s effectiveness in classification of brain activity.

**Figure 7:**
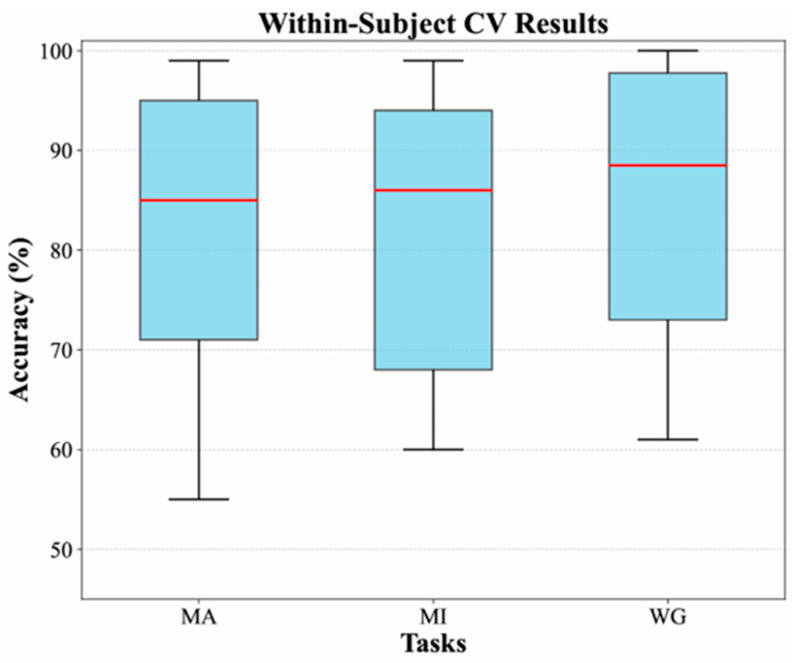
Box plots of classification accuracies from within-subject cross-validation.

Beyond average performance, the stability of the model’s performance across individual subjects is also crucial. Figure 7 provides a clear visualization of this performance distribution at the subject level using box plots. The box plots show that the model’s median accuracy on the MI and WG tasks is higher than that on the MA task, but there is also greater individual variability, as indicated by the larger interquartile range (IQR). In contrast, the model demonstrates more stable and consistent performance on the MA task.

### 3.5. Ablation Studies

To thoroughly investigate the contributions of each core component in BiSTF-Net and validate the necessity of its design, a series of detailed ablation studies were performed. Various model variants were constructed by removing or replacing key modules, and their performance was evaluated across all three tasks. These variants include: (1) replacement of the EEG input with a temporally shuffled version (temporally shuffled EEG); (2) removal of the spatial mapping process for both EEG and fNIRS inputs (w/o spatial mapping); (3) removal of the entire fNIRS branch (w/o fNIRS branch) or EEG branch (w/o EEG branch) to establish unimodal performance; (4) removal of the bidirectional cross-modal guidance module (w/o Bi-CMG); (5) removal of the adaptive temporal alignment module (w/o ATA); and (6) removal of the symmetric cross-attention fusion module (w/o SCAF). The detailed experimental results are provided in Table 1.

First, the results from the newly added ablation studies underscore the critical importance of specific data representation strategies in our BiSTF-Net framework. The replacement of EEG input with a temporally shuffled version (temporally shuffled EEG) resulted in significant performance drops, with average accuracies of 72.50% (MA), 70.00% (MI), and 71.67% (WG). This substantial degradation clearly demonstrates that preserving the genuine temporal dynamics of EEG signals is crucial for their effective use as high-temporal-resolution anchors, validating the necessity of accurate temporal information for cross-modal alignment. Similarly, removing the spatial mapping process (w/o spatial mapping) led to noticeable performance declines, with average accuracies of 68.33% (MA), 67.67% (MI), and 71.33% (WG). This indicates that the strategic transformation of multi-channel signals into a topologically rich 16 × 16 spatial grid, facilitated by interpolation, is fundamental for our deep learning architecture (particularly the 3D CNNs) to effectively extract and leverage complex spatial features. These findings collectively reinforce that our integrated data representation and processing pipeline are integral to the BiSTF-Net’s ability to overcome spatio-temporal heterogeneity and achieve superior performance.

Second, the results clearly demonstrate the fundamental advantage of multimodal fusion. Across all tasks, the performance of the complete BiSTF-Net model significantly outperforms any unimodal baseline. These baselines, derived by ablating the entire branch of the other modality within our framework, represent the performance ceiling of relying on a single data source. Specifically, across the MA, MI, and WG tasks, the proposed model improves accuracy by a range of 5.30% to 10.55% compared to the w/o EEG branch model, and by a substantial 16.04% to 20.67% compared to the w/o fNIRS branch model. This clearly illustrates that effectively combining the complementary information from both modalities can significantly overcome the inherent limitations of relying on a single modality.

Third, the ablation analysis of the three core modules highlights their individual, indispensable roles. Removing the Bi-CMG module results in a substantial performance drop, with accuracy decreasing by 4.52% to 6.64% across all tasks, demonstrating that bidirectional guidance during the early stages of feature extraction is crucial for learning high-quality synergistic features. The removal of the ATA module causes the most significant performance degradation among all the ablation experiments, with accuracy dropping by 4.52% to 7.00% across the three tasks., respectively. This strongly supports the notion that adaptive temporal alignment is the core solution to the critical bottleneck of fNIRS signal delay, making ATA the most crucial component for the model’s superior performance. Lastly, removing the SCAF module leads to a notable performance decline, with a decrease ranging from 2.33% to 5.69%, confirming the significant advantage of the proposed symmetric deep fusion framework over simpler alternatives.

### 3.6. Comparative Experiments

To comprehensively evaluate the performance of BiSTF-Net, we conducted comparative experiments with several state-of-the-art methods for EEG-fNIRS cognitive task classification, as shown in Table 2. These methods were selected because they represent recent advances in multimodal deep learning methods and have demonstrated strong performance in related tasks. For all baseline methods, we either utilized the publicly available code provided by the authors or reimplemented the algorithms based on the descriptions in their respective publications. All methods were evaluated using the same datasets, training procedures, and evaluation metrics to ensure a fair and consistent comparison.

Table 2 presents a detailed performance comparison between BiSTF-Net and six representative baseline methods. The results clearly demonstrate that BiSTF-Net achieves superior classification performance across all three cognitive tasks. First, BiSTF-Net consistently achieves the best average accuracy across all three tasks, reaching 83.33% for MA, 82.09% for MI, and 84.99% for WG. Second, comparing BiSTF-Net to the second-best performing method, E-FNet, BiSTF-Net shows significant improvements: specifically, a 3.97% gain in average accuracy on the MA task (83.33% vs. 79.36%), and a 3.23% gain in accuracy on the WG task (84.99% vs. 81.76%). Third, even on the Motor Imagery (MI) task, where some baseline models might exhibit competitive performance, BiSTF-Net maintains its leading position. While E-FNet achieves an accuracy of 80.52%, BiSTF-Net outperforms it in both recall and F1-score (82.55% and 81.59% respectively), demonstrating a more robust ability to identify positive instances and achieve overall competitive performance. These findings collectively validate the effectiveness of our proposed decoupled spatio-temporal fusion framework.

### 3.7. Visualization

To further explore the underlying mechanisms behind BiSTF-Net’s performance advantages, a visualization analysis was conducted from several perspectives.

**Figure 8:**
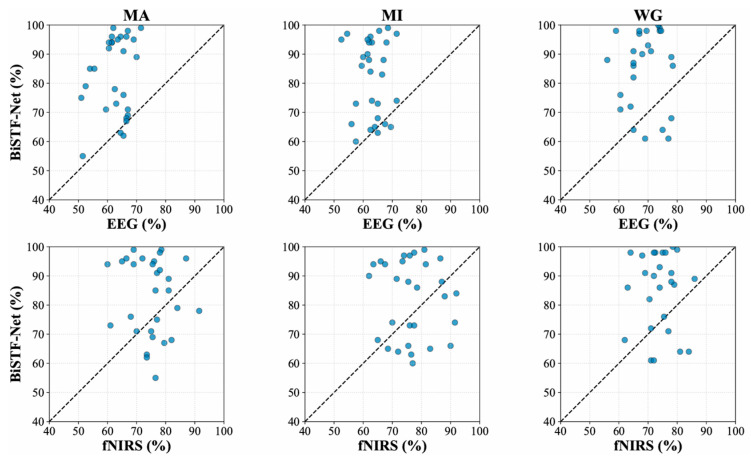
Scatter plots of classification accuracy for each subject. Each scatter point corresponds to an individual subject, with the horizontal axis representing unimodal accuracy and the vertical axis denoting BiSTF-Net’s accuracy.

To validate the effectiveness of multimodal fusion at the individual subject level, the classification accuracies of the BiSTF-Net and unimodal models were compared, as shown in Figure 8.

The scatter plots clearly indicate that, across all three tasks, the vast majority of data points lie above the diagonal line, intuitively demonstrating the general superiority of BiSTF-Net at the individual level. Notably, the performance improvement is most significant for subjects where the unimodal models performed poorly, emphasizing the unique value of this fusion strategy in challenging scenarios where information from a single modality is insufficient.

**Figure 9:**
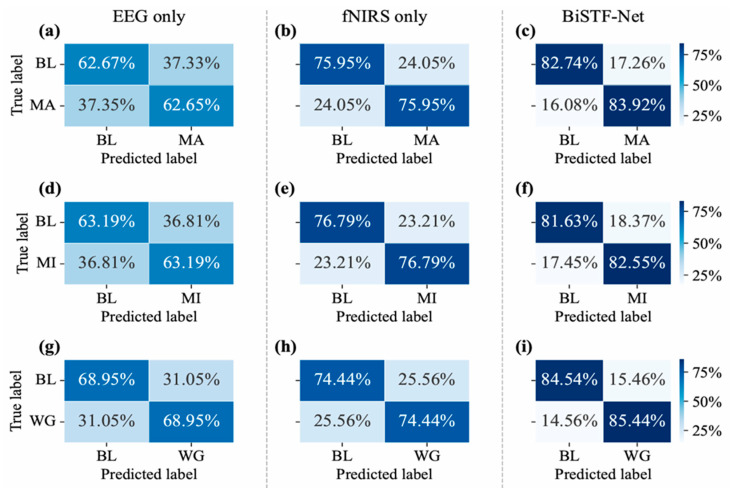
Confusion matrices for the three cognitive tasks of mental arithmetic (MA, **top** row), motor imagery (MI, **middle** row), and word generation (WG, **bottom** row) using the following methods: (**a**,**d**,**g**) EEG-only, (**b**,**e**,**h**) fNIRS-only, and (**c**,**f**,**i**) the proposed BiSTF-Net (BL: Baseline).

The confusion matrix analysis provides an intuitive demonstration of how multimodal fusion corrects the classification biases of unimodal approaches, as shown in Figure 9. A cross-modal comparison reveals the inherent limitations of the unimodal models, which consistently exhibit lower recall for the task-specific classes. Specifically, for the MA, MI, and WG tasks, the EEG-only model (corresponding to the w/o fNIRS branch ablation) achieved recalls of only 62.65%, 63.19%, and 68.95%, respectively. While the fNIRS-only model (corresponding to the w/o EEG branch ablation) performed better, its recalls were still limited to 75.95%, 76.79%, and 74.44%. In stark contrast, our proposed BiSTF-Net significantly boosts performance across all tasks, achieving substantially higher recalls of 83.92% (MA), 82.55% (MI), and 85.44% (WG). This comprehensive improvement strongly suggests that, after temporal alignment by the ATA, the stable spatial information from fNIRS effectively compensates for the limitations of the EEG signal, leading to more robust and accurate judgments.

## 4. Discussion

This study successfully developed and validated BiSTF-Net, a novel deep learning framework designed to address the core challenge of spatio-temporal heterogeneity in multimodal EEG-fNIRS neuroimaging. The central finding of this research is that employing a decoupled, multi-stage strategy for spatio-temporal fusion significantly enhances the classification performance of multimodal neural signals. The success of BiSTF-Net can be largely attributed to the synergistic interaction of its three core components, which span early guidance, mid-stage alignment, and late-stage deep fusion. This end-to-end decoupled design is what fundamentally drives the model’s superior performance.

The superior performance demonstrated by BiSTF-Net over existing methods like STA-Net and E-FNet stems from its advanced decoupled fusion strategy. Compared to STA-Net, BiSTF-Net introduces two key advancements. First, STA-Net uses unidirectional guidance from EEG to fNIRS, whereas BiSTF-Net’s Bi-CMG module facilitates bidirectional guidance, ensuring a more comprehensive exchange of information and early synergistic feature learning. Second, STA-Net directly uses aligned features for classification, resulting in a simpler fusion mechanism. In contrast, BiSTF-Net features a dedicated SCAF module for symmetric deep fusion, enabling a more thorough integration of high-level features from both modalities. When compared to other advanced models like E-FNet, BiSTF-Net’s main advantage lies in its three-stage decoupled fusion strategy. Many existing models attempt to process spatio-temporal features through a single complex network, trying to solve both alignment and fusion issues simultaneously. However, BiSTF-Net breaks down this complexity into clear stages: Bi-CMG handles early interaction, ATA specializes in temporal alignment, and SCAF performs late-stage deep fusion. This approach ensures more defined tasks and clearer optimization objectives for each module, resulting in superior overall performance by effectively addressing the challenges of spatio-temporal heterogeneity in a structured manner.

The implications of BiSTF-Net extend beyond benchmark performance and hold significant relevance for neurophysiology, cognitive neuroscience, and neurorehabilitation. While task-versus-baseline classification might seem straightforward, as noted by the reviewer, our proposed architecture offers a robust foundation for more nuanced analysis. The integration of high-temporal-resolution EEG and high-spatial-resolution fNIRS features, coupled with precise temporal alignment via the ATA module, enables a more comprehensive understanding of the spatio-temporal dynamics underlying cognitive processes. Furthermore, BiSTF-Net provides a valuable tool for neurorehabilitation applications, such as brain–computer interfaces (BCIs) for neuroprostheses. Unlike simple task detection, discriminating between stimulus types or distinct motor intents (e.g., left vs. right hand motor imagery) presents a significantly more complex challenge. Our framework’s ability to effectively fuse complementary information from both modalities, as demonstrated in the MI task, provides a robust solution for enhancing BCI performance in real-world neurorehabilitation scenarios. This decoupled approach offers a reliable method for identifying subtle neural differences required for precise neuroprosthetic control and offers a path toward developing more sophisticated BCI systems that move beyond simple task detection.

In summary, the superior performance of BiSTF-Net is fundamentally rooted in its sophisticated design philosophy. The decoupled architecture, featuring Bi-CMG, ATA, and SCAF modules, offers an effective and generalizable solution for tackling heterogeneous signal fusion challenges in neuroengineering.

## 5. Conclusions

This paper introduced and validated BiSTF-Net, a novel decoupled bi-directional spatio-temporal fusion network that effectively addresses the critical challenge of heterogeneity in multimodal EEG-fNIRS neuroimaging. Three key conclusions can be drawn from our comprehensive experiments. First, the proposed framework demonstrates superior classification performance, significantly outperforming existing baseline methods and confirming the efficacy of the fusion strategy at both the group and individual levels. Second, the model’s success is fundamentally rooted in its sophisticated decoupled design. Ablation studies confirmed the indispensable contributions of each core module, particularly highlighting the central role of adaptive temporal alignment in overcoming the critical bottleneck of fNIRS signal delay. Finally, visualization analyses revealed the model’s interpretable mechanisms, demonstrating how the fusion of multimodal information effectively compensates for the limitations of single-modality signals. This work contributes not only a powerful and reliable new framework for high-performance multimodal neural classification but also offers insightful design principles for tackling broader heterogeneous signal fusion challenges.

## Figures and Tables

**Table 1:** Ablation study of BiSTF-Net.

Task	Method	Accuracy (%)	Precision (%)	Recall (%)	F1-Score (%)
MA	temporally shuffled EEG	72.50	65.43	72.50	64.85
	w/o spatial mapping	68.33	65.58	72.17	70.57
	w/o EEG branch	75.95	73.44	75.95	71.56
	w/o fNIRS branch	62.66	58.44	62.65	55.70
	w/o Bi-CMG	77.74	74.43	77.74	73.20
	w/o ATA	76.69	75.13	76.69	72.85
	w/o SCAF	79.76	78.14	79.76	76.08
	BiSTF-Net (ours)	83.33	84.59	83.92	83.30
MI	temporally shuffled EEG	70.00	68.75	73.51	73.59
	w/o spatial mapping	67.67	61.49	69.13	65.63
	w/o EEG branch	76.79	74.00	76.79	72.64
	w/o fNIRS branch	63.19	60.43	63.19	56.36
	w/o Bi-CMG	77.57	74.74	77.57	73.18
	w/o ATA	77.57	76.00	77.57	73.80
	w/o SCAF	79.76	78.23	79.76	76.01
	BiSTF-Net (ours)	82.09	81.26	82.55	81.59
WG	temporally shuffled EEG	71.67	71.59	77.03	78.75
	w/o spatial mapping	71.33	67.04	70.52	69.29
	w/o EEG branch	74.44	76.84	74.44	73.26
	w/o fNIRS branch	68.95	70.44	68.95	66.40
	w/o Bi-CMG	78.35	80.47	78.35	77.61
	w/o ATA	77.99	79.75	77.99	77.35
	w/o SCAF	79.30	81.30	79.30	78.75
	BiSTF-Net (ours)	84.99	85.37	85.44	84.96

**Table 2:** Performance comparison of different methods.

Task	Method	Accuracy (%)	Precision (%)	Recall (%)	F1-Score (%)
MA	E-FNet	79.36	80.14	79.75	79.36
	STA-Net	74.95	69.32	74.95	69.17
	ECA-FusionNet	66.33	67.32	66.69	66.38
	ST2A	75.47	75.04	75.47	71.86
	M2NN	64.91	63.33	64.91	60.20
	EF-Net	67.95	59.81	67.95	59.92
	BiSTF-Net (ours)	83.33	84.59	83.92	83.30
MI	E-FNet	80.52	81.77	80.52	80.66
	STA-Net	71.00	71.36	71.45	71.62
	ECA-FusionNet	66.84	67.45	66.12	66.71
	ST2A	75.28	74.42	75.28	71.28
	M2NN	65.31	64.64	65.31	60.84
	EF-Net	68.55	61.04	68.55	60.75
	BiSTF-Net (ours)	82.09	81.26	82.55	81.59
WG	E-FNet	81.76	75.54	78.66	76.71
	STA-Net	79.92	78.68	78.58	76.47
	ECA-FusionNet	81.00	78.92	77.13	76.09
	ST2A	79.43	78.38	78.52	75.93
	M2NN	74.47	71.40	70.96	69.45
	EF-Net	78.36	76.90	75.39	73.60
	BiSTF-Net (ours)	84.99	85.37	85.44	84.96

## Data Availability

The datasets used in this study are publicly available at https://doc.ml.tu-berlin.de/simultaneous_EEG_NIRS/ (accessed on 14 October 2025) and https://doc.ml.tu-berlin.de/hBCI/ (accessed on 14 October 2025), and the source code for our proposed model and analysis is also publicly available at https://github.com/zc701/BiSTF-Net (accessed on 20 January 2026).
